# The Mycobacterium abscessus *mbtE* Analog MAB_2122 Is, in Contrast to the *mbtE* Analog MAB_2248c, Dispensable for Normal Growth in Low-Iron Conditions

**DOI:** 10.1128/spectrum.05160-22

**Published:** 2023-01-25

**Authors:** Mark Foreman, Daniel Barkan

**Affiliations:** a Koret School of Veterinary Medicine, The Robert H. Smith Faculty of Agriculture, Food and Environment, The Hebrew University of Jerusalem, Rehovot, Israel; Johns Hopkins University School of Medicine

**Keywords:** *Mycobacterium abscessus*, iron, mycobactin, *mbtE*, iron acquisition

## LETTER

**FIG 1:**
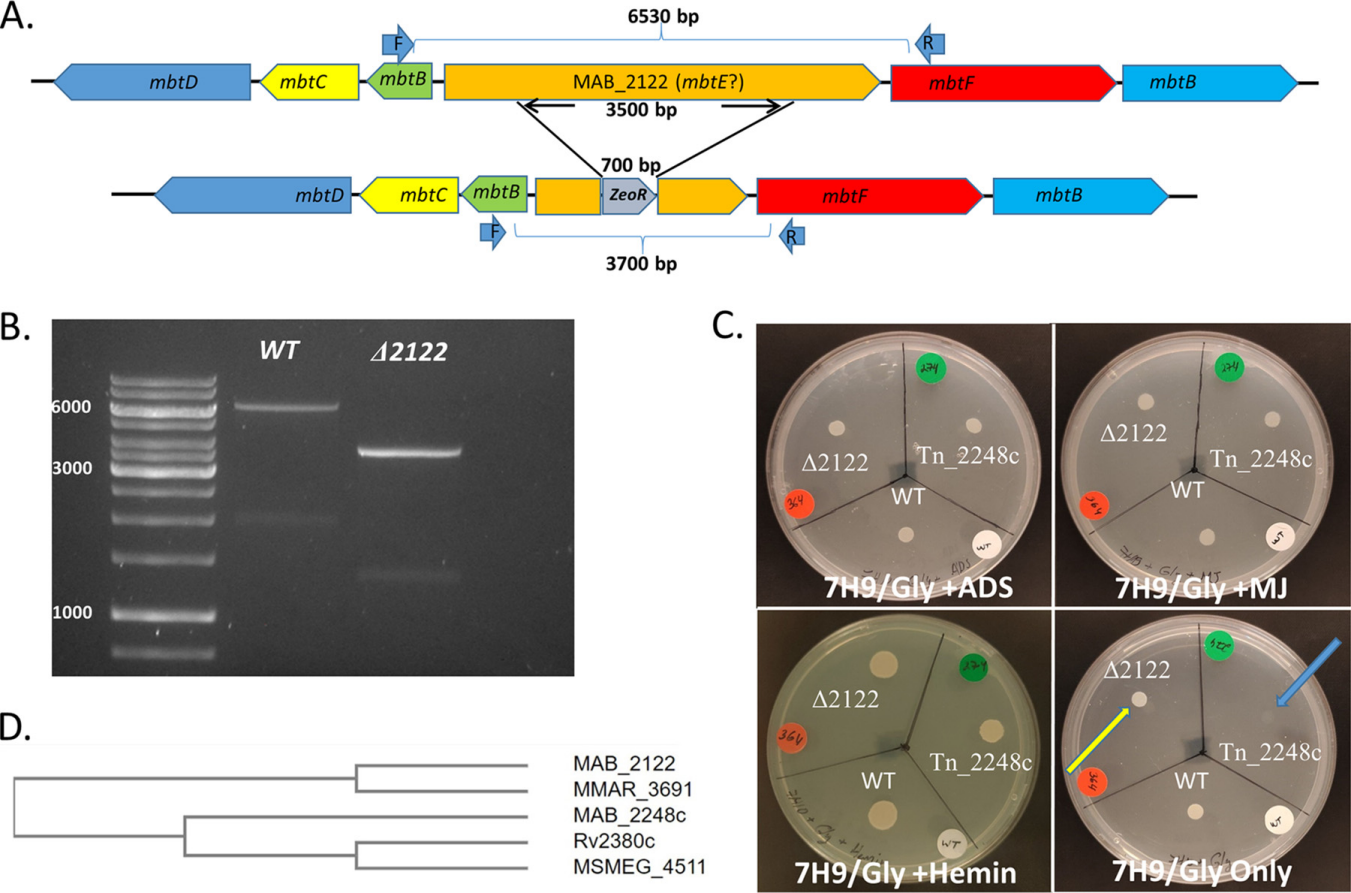
(A) Genomic organization of MAB_2122 and the surrounding *mbt* genes (top) and the resulting genomic organization when the middle 3,500 bp of MAB_2122 are replaced with the *Zeo*^r^ gene (bottom). PCR performed using forward (F; 5′-AACGGCGGTCATCGTTGTGGCGAGG-3′) and reverse (R; 5′-CTAGGGACCCTCCTGCCTGGTAGAC-3′) primers yielded an ~6,500-bp product in WT cells and a 3,700-bp product in a correct deletion mutant (note that both MAB_2121c [green] and MAB_2124 [light blue] are annotated as *mbtB* in Mycobrowser, with MAB_2121c annotated as *mbtB*/thioesterase). (B) PCR products of the F and R primers in WT cells and a deletion mutant candidate were compared by electrophoresis, confirming the deletion. (C) 5,000 CFU of WT, mDB274 (Tn_2248c), and mDB364 (Δ2122) was spotted onto 7H9-glycerol (Gly) agar plates supplemented with albumin-dextrose (top left), hemin (bottom left), mycobactin J (top right), or no supplementation (bottom right) and kept at 37°C for up to 7 days. The yellow arrow indicates mDB364; the blue arrow indicates mDB274. (D) Phylogenetic tree of MbtE analogs (by amino acid sequence) from M. tuberculosis, Mycobacterium smegmatis, M. abscessus (both 2122 and 2248c), and Mycobacterium marinum.

Recently, we characterized MAB_2248c as an analog of the *mbtE* gene of Mycobacterium tuberculosis (1). A transposon (Tn) disruption mutant of MAB_2248c grew slowly on media relatively poor in iron, and this slow growth phenotype was salvaged by mycobactin J (MJ), hemin, blood, and also by albumin. The transcription of MAB_2248c (the part before the transposon insertion) was induced in the transposon mutant, as well as in wild-type (WT) bacteria when grown in iron-poor conditions. M. tuberculosis, as well as most other mycobacteria, has one copy of *mbtE* (2, 3). Interestingly, in Mycobacterium abscessus, there are 2 genes with similarity to the *mbtE* of M. tuberculosis, and both are annotated as “*mbtE*” in databases such as Mycobrowser (https://mycobrowser.epfl.ch/). Whereas MAB_2248c has 51.6% similarity to M. tuberculosis *mbtE*, the other gene, MAB_2122, has 55% similarity—although the similarity between the two is only 42.8%. Genes around MAB_2122 are also designated *mbt* genes (Fig. 1A). The transcription of MAB_2122 did not increase in the MAB_2248c transposon mutant, nor in WT bacteria when grown in iron-poor conditions (1).

All this raised questions about the role of MAB_2122—whether this was a functional *mbtE* analog, and whether these two genes were redundant or acted in parallel. To test whether MAB_2122 had a role in normal growth and iron acquisition in M. abscessus, we constructed a targeted deletion mutant of this gene. This was done by electroporating a linear deletion construct, with the flanking regions of MAB_2122 and the Zeocin resistance gene (*Zeo*^r^) between them, into M. abscessus (ATCC 19977) bearing plasmid pJV53, used for recombineering (4). Transformants were plated onto 7H9-glycerol-ADS (albumin-dextrose)-MJ agar plates (to ensure that even a strict iron auxotroph would grow) supplemented with Zeocin (66 μg/mL). One of the resulting colonies was examined using PCR, which yielded a 6,530-bp product in WT, but only a 3,700-bp product in the mutant, where most of the MAB_2122 gene was replaced by *Zeo*^r^ (Fig. 1A shows a graphic representation; Fig. 1B shows the PCR electrophoresis). The colony was thus confirmed to be a MAB_2122 deletion mutant and was named mDB364. The previously created MAB_2248c transposon mutant was named mDB274. To examine whether the ΔMAB_2122 mutant was an iron auxotroph and was dependent on iron or mature mycobactin J (as is mDB274), we grew WT, mDB274, and mDB364 to an optical density at 600 nm (OD_600_) of 0.2, washed them twice in phosphate-buffered saline (PBS), diluted them to a concentration of ~2,500 CFU/μL, and spotted 2 μL onto 7H9-glycerol plates supplemented with either ADS, mycobactin J, hemin, or no supplementation (Fig. 1C). All three isolates grew well on plates supplemented with ADS, MJ, or hemin. On the plate with 7H9-glycerol only, the MAB_2248c Tn mutant grew poorly (if at all) (Fig. 1C, bottom right, blue arrow)—as expected. In contrast, the ΔMAB_2122 mutant did not exhibit any appreciable growth retardation (yellow arrow) compared to the WT.

We then analyzed by comparison the sequences of all three genes, MAB_2248c, MAB_2122, and the M. tuberculosis *mbtE* (Rv2380c). Although all three genes appear to share significant homology, and by length MAB_2122 is actually closer to Rv2380c than MAB_2248c, when we used the Clustal Omega online tool for protein sequence homology (5), the phylogenetic tree showed that the MAB_2122 protein was further away from the MbtE of M. tuberculosis than MAB_2248c (Fig. 1D).

Additionally, we performed a promoter prediction analysis on the (−250) bp sequence for MAB_2122, MAB_2248c, and Rv2380c, using the online tool PromoterHunter (http://www.phisite.org/main/index.php?nav=tools&nav_sel=hunter). In both MAB_2248c and Rv2380c, the program predicted the existence of a promoter in the (−45) to (−10) bp region, with high homology between the two predicted promoters. In contrast, in MAB_2122, no promoter was predicted at close proximity to the start codon, and the closest predicted promoters were found as far away as (−110) bp from the start codon. Of note, if indeed MAB_2122 has a poorly defined promoter (if any), this may also explain why in our previous work (1), we did not find a change in the transcription level of MAB_2122, nor of its downstream genes (*mbtF*, *mbtB*) under iron-limiting conditions.

To summarize, M. abscessus, in contrast to most other mycobacteria, has two analogs of *mbtE*. Whereas the inactivation of one of them (MAB_2248c) resulted in substantial growth retardation on iron-poor media, the deletion of MAB_2122 did not appear to have the same effect, and bacteria grew apparently normally on media that did not support the growth of Tn_2248c. The role of MAB_2122 could therefore be either negligible or completely compensated by MAB_2248c, whereas MAB_2122 could not compensate for the loss of MAB_2248c to any substantial effect. In our previous experiment, iron deprivation caused an increase in the transcription of MAB_2248c but had no effect on the transcription of MAB_2122, which is in agreement with the apparent lack of an obvious promoter region for this gene. Whether the duplication of *mbtE* resulted in one active and one inactive enzyme or whether the differences were related to regulation (and activity of MAB_2122 under specific conditions that we did not identify) remains unclear. Coevolution of transcriptional and enzymatic regulation in what appears to be duplicate genes has been described before (6). However, it appears that at least under standard laboratory conditions, the role of MAB_2122 in the growth of M. abscessus is, in contrast to that of MAB_2248c, negligible.
